# Self-Esteem, Social Comparison, and Facebook Use

**DOI:** 10.5964/ejop.v14i4.1592

**Published:** 2018-11-30

**Authors:** Elisa Bergagna, Stefano Tartaglia

**Affiliations:** a Department of Psychology, University of Turin, Turin, Italy; Department of Psychology, Webster University Geneva, Geneva, Switzerland; University of Western Ontario, London, Canada

**Keywords:** Facebook use, self-esteem, social comparison, gender differences, social media

## Abstract

Facebook use is very popular among young people, but many open issues remain regarding the individual traits that are antecedents of different behaviours enacted online. This study aimed to investigate whether the relationship between self-esteem and the amount of time on Facebook could be mediated by a tendency towards social comparison. Moreover, three different modalities of Facebook use were distinguished, i.e., social interaction, simulation, and search for relations. Because of gender differences in technology use and social comparison, the mediation models were tested separately for males and females. Data were collected by means of a self-report questionnaire with a sample of 250 undergraduate and graduate Italian students (mean age: 22.18 years). The relations were examined empirically by means of four structural equation models. The results revealed the role of orientation to social comparison in mediating the relations between low self-esteem and some indicators of Facebook use, i.e., daily hours on Facebook and the use of Facebook for simulation. For females, the use of Facebook for social interaction was directly influenced by high self-esteem and indirectly influenced by low self-esteem. Globally, the dimension of social comparison on Facebook emerged as more important for females than for males.

The development of the World Wide Web has completely changed the way people communicate and spread information (Krämer & Winter, 2008). Through social media, people can share personal information with a broader community of people by posting photos, videos and blogs (Kaplan & Haenlein, 2010). Social networking sites such as Myspace and Facebook are widely used by adolescents, young adults, and people over 30 years old (Wilson, Gosling, & Graham, 2012). Considering all the friend-networking sites available online, Facebook seems to be the most utilized in the world, with over 1 billion visitors per month (Facebook Press Room, 2017). Several studies linked Facebook use to positive effects, such as the satisfaction of users’ needs for feelings of self-worth and self-integrity (Toma & Hancock, 2013), an improvement in the quality of existing friendships (Valkenburg & Peter, 2009), and the perception of greater subjective well-being (Kim & Lee, 2011). However, other scholars have found the opposite results, providing evidence of negative consequences related to Facebook use. For instance, excessive use of social media, sometimes recognized as Internet addiction, has been related to high levels of depressive symptoms (Marino, Hirst, Murray, Vieno, & Spada, 2017; Morrison & Gore, 2010). Moreover, some scholars found that people who used Facebook more frequently have reported that others are happier and live better than they do themselves (Chou & Edge, 2012). However, these negative psychological outcomes are mainly related to the way people use Facebook rather than to the social network itself. For most people, this social medium does not have positive or negative consequences, but for other individuals, Facebook use may be a maladaptive way of escaping from problems or relieving a dysphoric mood (Cash, Rae, Steel, & Winkler, 2012). For this reason, it becomes important to investigate individual traits that can predict harmful online behaviours so they can be prevented.

## Facebook and Self-Esteem

In psychology, self-esteem is defined as the positive or negative evaluation of the self, and many theories have suggested that maintaining or raising it is a basic human need (Weiten, 2004). Regarding the relationship between self-esteem and Facebook use, scholars have found contradictory results. Most studies (Kalpidou, Costin, & Morris, 2011; Mehdizadeh, 2010; Tazghini & Siedlecki, 2013) found that people with lower self-esteem spend more time on Facebook. Some authors have interpreted this finding using social compensation theory. Social compensation theory, or the poor-get-richer hypothesis, states that people who experience difficulties in social relations, such as individuals with low self-esteem, socially anxious individuals, and introverts, are more motivated to use social networks in order to compensate for their unsatisfactory face-to-face interactions (McKenna, Green, & Gleason, 2002). An opposite perspective is social enhancement, or the rich-get-richer hypothesis. According to this theory, for more sociable young people, there are added benefits from expanding communication through social media: socially active people, with high levels of self-esteem, are more inclined to utilize friend-networking sites as a means to extend their social network further (Kraut et al., 2002). However, few studies have found evidence that people with a positive self-evaluation spend more time on Facebook (Ghosh & Dasgupta, 2015). Instead, other studies found no relation between self-esteem and Facebook use (Skues, Williams, & Wise, 2012). As some scholars have suggested, the literature may be inconsistent because studies have considered Facebook as a whole without distinguishing between different modalities of use or motivations to utilize the social network (Rae & Lonborg, 2015; Tartaglia, 2016). Generally, people who communicate easily with others face to face have less need to spend a great deal of time online, but when considering the specific ways Facebook can be used, a different result can be found (Wilson, Fornasier, & White, 2010). A recent study (Tartaglia, 2016) has shown three different modalities of using Facebook, i.e., social interaction, simulation, and search for relations. The first is the use of the social network for interaction with friends and for self-expression. High self-esteem was found to be a predictor of this modality of using Facebook. In contrast, people who use Facebook to simulate a self-image online that differs from reality had low self-esteem. Finally, the use of Facebook to seek new relations, i.e., meeting people, was not related to self-esteem.

### Social Comparison

Social comparison takes place when individuals compare themselves with others on abilities and personal characteristics. Festinger (1954) was the first to theorize that comparing oneself to others is a human need essential for acquiring information about the self. As some authors noted (Gilbert, Price, & Allan, 1995), the need to compare oneself to others is phylogenetically very old and biologically very powerful; however, individuals differ greatly in the extent to which they tend to confront each other (Gibbons & Buunk, 1999). Many scholars (Buunk & Gibbons, 2007; Steil & Hay, 1997; Wheeler, 2000) have suggested that certain types of individuals may be more inclined to compare than others, hypothesizing that the tendency to engage in social comparisons may be a personality characteristic. The “typical” individual with a high tendency to engage in social comparison is characterized by certain features, such as an increased sensitivity to the behaviour of others and a degree of uncertainty about the self, along with a great need to reduce this uncertainty and improve her/his self-worth (Gibbons & Buunk, 1999). Individuals with low self-esteem, whose self-concepts are inconstant and uncertain, are particularly interested in making social comparisons (Buunk & Gibbons, 2007). This tendency towards social comparison may be facilitated by modern technologies, which have transferred many social relations from the private to the public sphere, exposing people to a continual flow of information (Subrahmanyam & Greenfield, 2008). Specifically, people on Facebook may compare themselves with others on the number of likes and the types of comments that other people have posted to their statuses and photos; moreover, a friend’s status or photos may trigger specific social comparisons, which can make people feel better or worse (Steers, Wickham, & Acitelli, 2014). Researchers have mainly focused on the consequences of Facebook social comparisons (Feinstein et al., 2013; Krasnova, Wenninger, Widjaja, & Buxmann, 2013; Nesi & Prinstein, 2015): their results have shown that social comparison on Facebook may predict depression, rumination and lower life satisfaction. However, some authors (Nesi & Prinstein, 2015) emphasized the hypothesis that depressive symptoms precede and predict social comparisons on Facebook, stating that individuals with low self-esteem who are prone to depression are more likely to spend time on Facebook engaging in higher levels of social comparison. This point of view seems to suggest that a greater tendency towards social comparison might increase the use of this social network, motivated by the search for information about others.

In summary, social comparison theory suggests that self-esteem influences the tendency to compare oneself with others, which in turn would influence the use of Facebook because it may be a tool for social comparison. Although some scholars have suggested this relation (Nesi & Prinstein, 2015), no study to our knowledge has examined the role of social comparison orientation in influencing the use of Facebook, neither the overall use nor the specific modalities of use. We aimed to empirically test this theoretical model. Considering the different modalities of using Facebook, we can make some specific assumptions. For people whose evaluation of the self is mainly based on the perceived opinions of others (i.e., social comparison), self-expression can become a way to reduce uncertainty about the self by eliciting positive feedback from others (Nesi & Prinstein, 2015). Moreover, individuals with a strong social comparison orientation may use Facebook for simulation so that they can gain benefits from comparisons and increase their self-esteem. These people are worried about image and very interested in what others think (Buunk & Gibbons, 2007). Finally, the use of Facebook to search for relations should not be related to a social comparison tendency because this modality seems mainly to refer to the search for a romantic partner (Tartaglia, 2016).

### Current Research

Despite the contradictory results concerning the relationship between self-esteem and Facebook use, most research (Kalpidou et al., 2011; Tazghini & Siedlecki, 2013) has demonstrated that people who spend more time on Facebook are individuals with lower self-esteem because they need to compensate for their relational face-to-face difficulties. Additionally, individuals with low self-esteem, whose self-concepts are particularly uncertain, are especially interested in social comparison (Buunk & Gibbons, 2007), and this tendency might determine greater use of Facebook, since this social network makes it especially easy for people to compare themselves to others (Nesi & Prinstein, 2015). Therefore, we aimed to investigate whether the relationship between self-esteem and the amount of time on Facebook would be mediated by a tendency towards social comparison. Moreover, we wanted to test this mediational model not only on the general use of Facebook but also on three specific modalities of using Facebook, that is, social interaction, simulation and search for relations (Tartaglia, 2016). Finally, we wanted to test the mediation model for males and females separately, since previous research has found gender differences in technology use and social comparison (Nesi & Prinstein, 2015; Stefanone, Lackaff, & Rosen, 2011). Specifically, females seem to share more photos on social networking sites and spend more time maintaining their social networks than males (Rosen, Stefanone, & Lackaff, 2010). Women are more likely to compare themselves on the dimension of physical attractiveness based on online photos (Haferkamp & Krämer, 2011) and are more susceptible to comparing themselves to others on the dimension of self-expression, which they feel is personally relevant.

On these grounds, we expected the following:

- Self-esteem is negatively associated with the amount of time spent on Facebook (Kalpidou et al., 2011; Tazghini & Siedlecki, 2013) and with the use of the social networking site for simulation (Tartaglia, 2016). Self-esteem is positively related to the use of Facebook for social interaction, whereas it is not related to the use of Facebook to search for relations (Tartaglia, 2016).
- Self-esteem is negatively related to the social comparison orientation (Buunk & Gibbons, 2007).
- Because Facebook is a means for comparing oneself with others (Steers, Wickham, & Acitelli, 2014), social comparison orientation is related to time spent on Facebook, the use of Facebook for self-expression (i.e., the modality of social interaction) and simulation of a different self-image (i.e., the modality of simulation).
- Gender differences in technology use and social comparison (Nesi & Prinstein, 2015; Stefanone et al., 2011) lead to different results in the mediational models tested separately for males and females.

## Method

### Participants

The research sample included 250 university students (43.6% male and 56.4% female). The average age of the participants was 22.18 years (*SD* = 2.52). A psychology graduate student in collected the data for her master’s degree thesis. Participants attended different courses in the arts and sciences school of a public university. Participation was voluntary.

### Measures

We collected the data using a self-report questionnaire, which took approximately 20 minutes to complete. The following indicators were used in our analysis:

1. The Italian version of the Rosenberg Self-Esteem scale (Prezza, Trombaccia, & Armento, 1997), which is composed of 9 items assessing global self-esteem (e.g., “On the whole, I am satisfied with myself”). Items were rated on a 4-point Likert-type scale ranging from 1 (strongly disagree) to 4 (strongly agree). The scale showed good internal coherence (Cronbach’s α = .85).
2. The Iowa-Netherlands Comparison Orientation Measure (INCOM; Gibbons & Buunk, 1999), which included 11 items measuring participants’ tendency to socially compare themselves to others (e.g., “I always pay a lot of attention to how I do things compared with how others do things”). Items were rated on a 5-point Likert scale ranging from 1 (strongly disagree) to 5 (strongly agree). The scale showed good internal coherence (Cronbach’s α = .90).
3. The amount of time people spent on Facebook, which was assessed through one item asking participants “Approximately how many hours per day do you spend on Facebook?”
4. The modality of using Facebook scale (Tartaglia, 2016), including 19 items that presented different activities people can do on Facebook. The instructions for participants read “How often do you do the following things on Facebook?” and items were rated on a 4-point Likert-type scale ranging from 1 (never) to 4 (very often). The items belong to three subscales measuring different modalities of using Facebook: social interaction (e.g., “Comment on other people’s statuses, walls, or links”) consisting of 11 items (α = .79); simulation (e.g., “Hide some things about yourself that you don’t like) consisting of 5 items (α = .81); and search for relations (e.g., “Chat with people you don’t know”) consisting of 3 items (α = .72).
5. A brief list of socio-demographic items (i.e., gender, age).

### Data Analyses

After performing preliminary analyses, we investigated the hypothesized relationships by testing structural equation models. We tested the models simultaneously on males and females to investigate gender differences.

## Results

### Preliminary Analyses

Table 1 shows the mean scores of the measures used in the analyses. We calculated the means separately by gender group and tested the differences using t-tests. Females spent more time daily on Facebook than males. There are no other significant gender differences. Concerning the modality of Facebook usage, social interaction was the most frequent cited by participants. Table 2 shows the correlations among measures. Self-esteem correlated negatively with social comparison orientation, daily hours on Facebook, and simulation. Social comparison orientation correlated positively with all the Facebook use indicators. Each Facebook use indicator correlated positively with all the others except for daily hours on Facebook and search for relations, which did not correlate.

**Table 1:** Scale Scores for Males (n = 109) and Females (n = 141): Mean scores and t values

Variable	Mean scores		*t*
	Males	Females	
Self-esteem	3.13	3.08	0.72
Social comparison orientation	2.36	2.40	-0.44
Daily hours on Facebook	0.92	1.26	-2.28*
Modality of using Facebook			
Social interaction	1.96	2.00	-0.68
Simulation	1.71	1.83	-1.46
Search for relations	1.68	1.70	-0.30

*p < .05.

**Table 2:** Correlations Among Measures

Variable	1	2	3	4	5
1. Self-esteem					
2. Social comparison orientation	-.33**				
3. Daily hours on Facebook	-.29**	.23**			
Modality of using Facebook					
4. Social interaction	-.11	.48**	.36**		
5. Simulation	-.41**	.60**	.33**	.41**	
6. Search for relations	-.12	.21**	.09	.39**	.18**

*p < .05. **p < .01.

### Testing the Models

On the grounds of the abovementioned literature and the preliminary analyses, we tested four structural equations models predicting daily hours on Facebook and the three modalities of using Facebook, i.e., social interaction, simulation, and search for relations. All the models assumed the following: (a) Self-esteem influences both social comparison orientation and the Facebook use indicator; (b) Social comparison orientation influences the Facebook use indicator. We tested each model simultaneously on males and females, performing bootstrap analyses to investigate the indirect effect of self-esteem on the Facebook use indicator. We used a partial disaggregating approach (Bagozzi, 1993; Bagozzi & Edwards, 1998) to reduce the number of indicators for each latent variable and still allow for estimation of the measurement error. We randomly aggregated the items into three indicators for each scale, except for simulation, which was reduced to two indicators. As recommended (Hu & Bentler, 1998), we tested the model fit using different indexes to diminish the impact of their limits. We used χ^2^, CFI (Comparative Fit Index; Bentler, 1990), TLI (Tucker-Lewis Index; Tucker & Lewis, 1973) and RMSEA (root mean square error of approximation; Steiger, 1990).

**Figure 1:**
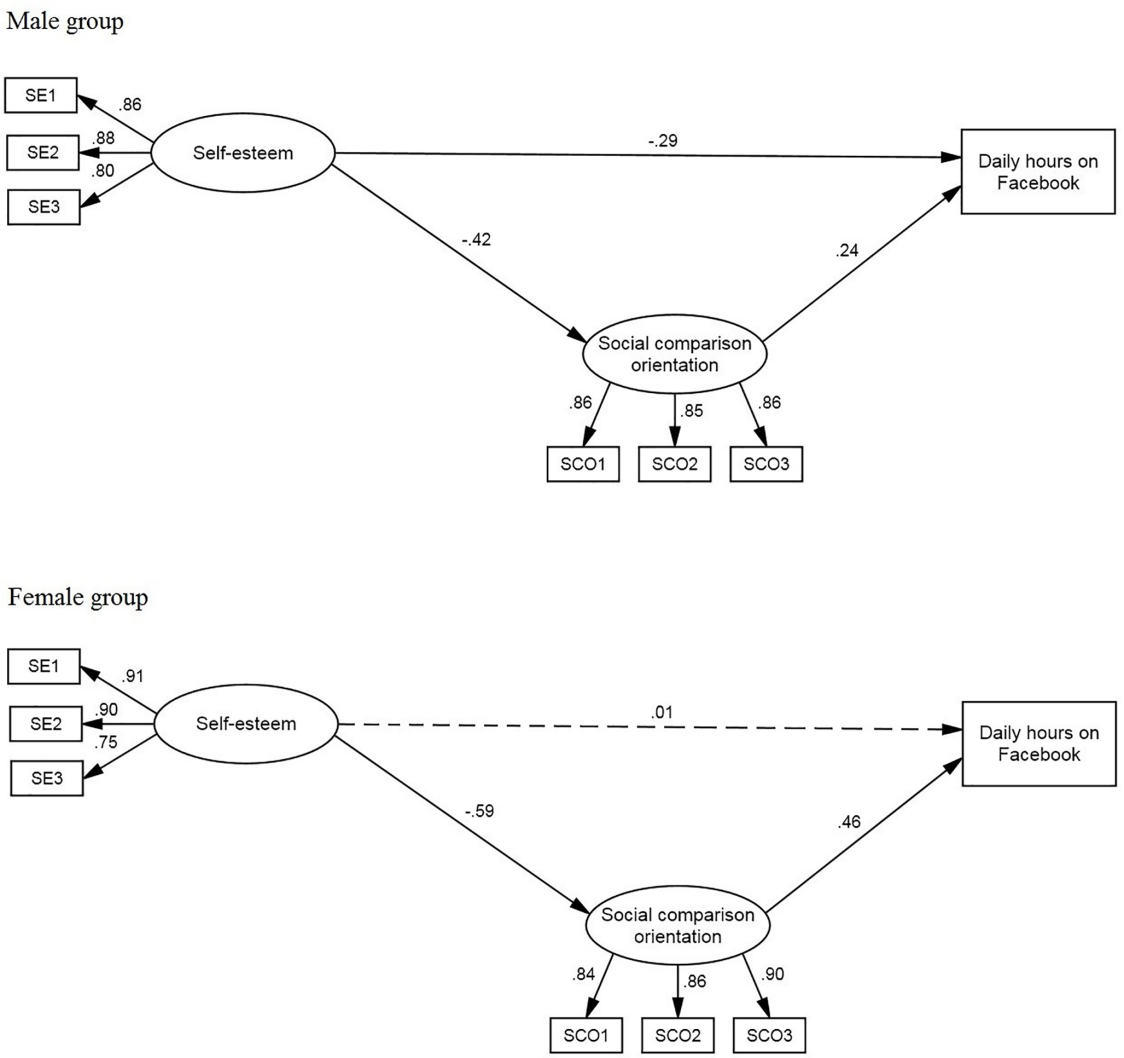
Model of the Prediction of Daily Hours on Facebook. Estimated parameters for gender groups: Standardized regression weights and variances. *Note.* Errors of the indicators and latent variables were omitted from the figure to make it easier to view.

The first model (see Figure 1) predicted daily hours on Facebook. The model fit was satisfactory: χ^2^(24) = 53.16, *p* < .01; χ^2^/gdl = 2.22; CFI = .97; TLI = .95; RMSEA = .070. For the male group, all the estimated parameters were significant. Self-esteem negatively influenced both social comparison orientation (β = -.42) and daily hours on Facebook (β = -.29). Social comparison orientation positively influenced daily hours on Facebook (β = .24). Bootstrapping showed an indirect effect of self-esteem on daily hours on Facebook (β = -.10; 95% CI = -.20 to -.05; *p* < .01; *SE* = .05). Considering the female group, self-esteem did not directly influence daily hours on Facebook. Self-esteem had a negative effect on social comparison orientation (β = -.59). Social comparison orientation positively influenced daily hours on Facebook (β = .46). Self-esteem had an indirect effect on daily hours on Facebook (β = -.27; 95% CI = -.37 to -.18; *p* < .01; *SE* = .06).

**Figure 2:**
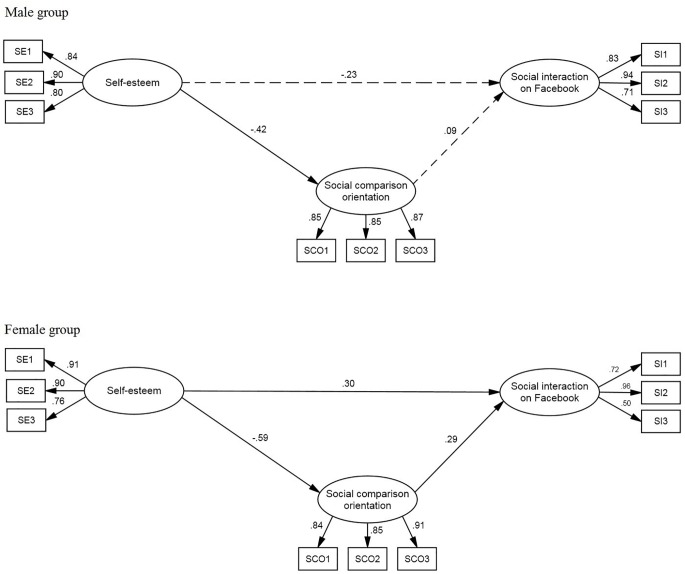
Model of Prediction of Social Interaction Use of Facebook. Estimated parameters for gender groups: Standardized regression weights and variances. *Note.* Errors of the indicators and latent variables were omitted from the figure to make it easier to view.

The second model (see Figure 2) predicted the social interaction use of Facebook. The model fit was satisfactory: χ^2^(48) = 107.11, *p* < .01; χ^2^/gdl = 2.23; CFI = .95; TLI = .93; RMSEA = .070. Concerning the male group, there were no significant effects on social interaction. The only significant parameter is the negative link between self-esteem and social comparison orientation (β = -.42). In contrast, all the parameters estimated for the female group were significant. Self-esteem negatively influenced social comparison orientation (β = -.59) and positively influenced social interaction (β = .30). Social comparison orientation positively influenced social interaction (β = .29).

**Figure 3:**
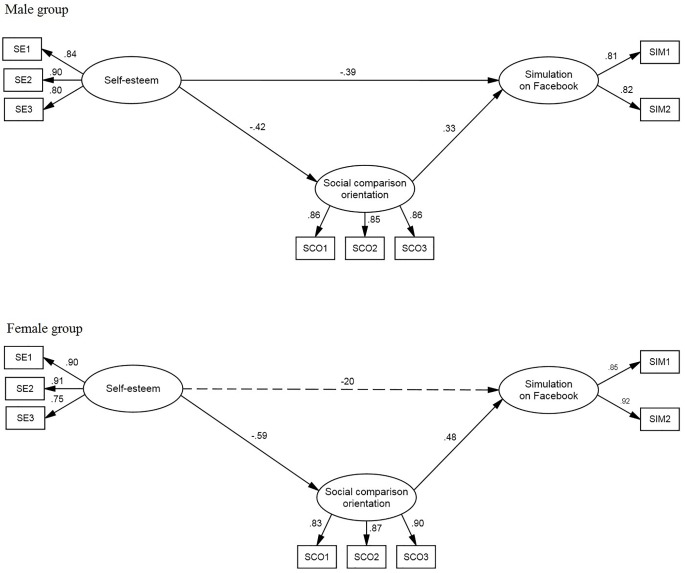
Model of Prediction of Simulation Use of Facebook. Estimated parameters on gender groups: Standardized regression weights and variances. *Note.* Errors of the indicators and latent variables were omitted from the figure to make it easier to view.

The third model (see Figure 3) predicted the simulation modality of use of Facebook. The model fit was satisfactory: χ^2^(34) = 83.72, *p* < .01; χ^2^/gdl = 2.46; CFI = .96; TLI = .94; RMSEA = .077.

All the parameters estimated for the male group were significant. Self-esteem negatively influenced both social comparison orientation (β = -.42) and simulation (β = -.39). Social comparison orientation positively influenced simulation (β = .33). Self-esteem had an indirect negative effect on simulation (β = -.14; 95% CI = -.26 to -.05; *p* <. 01; *SE* = .06). For the female group, the direct effect of self-esteem on simulation was not significant; the other relations were similar to those for the male group. Self-esteem negatively influenced social comparison orientation (β = -.59). Social comparison orientation positively influenced simulation (β = .48). Self-esteem had a negative indirect effect on simulation (β = -.29; 95% CI = -.43 to -.14; *p* < .01; *SE* = .09).

In the model predicting the search for relations, there were no significant influences on that modality of using Facebook.

## Discussion

The present study aimed at investigating whether the relationship between self-esteem and the amount of time on Facebook would be mediated by a tendency towards social comparison. Moreover, we wanted to distinguish between different kinds of use of the social network and referred to three specific modalities of using Facebook, i.e., social interaction, simulation, and search for relations. Finally, we wanted to test the possibility of different results when considering males and females separately.

Consistent with previous research (Nesi & Prinstein, 2015; Stefanone et al., 2011), we found that females spend more time daily on Facebook than males. However, we did not find gender differences concerning the different modalities of using Facebook.

In line with previous research (Kalpidou et al., 2011; Tazghini & Siedlecki, 2013), we found that self-esteem was negatively associated with the amount of time on Facebook, but only for males. We may interpret this result using the social compensation hypothesis, which maintains that people who are socially inept, such as individuals with low self-esteem, are less comfortable interacting with peers in face-to-face contexts and are strongly motivated to compensate for the deficiency by using online interactions (McKenna, Green, & Gleason, 2002). Consistent with previous research (Buunk & Gibbons, 2007), self-esteem was negatively related to the social comparison orientation, which in turn predicted a greater amount of time spent on Facebook: this path was found for both males and females. For females, social comparison orientation fully mediated the relation between self-esteem and the amount of time on Facebook because the direct path between the two variables was not significant. Females with low self-esteem seem to spend more time on Facebook in order to compare themselves to others and possibly increase their self-esteem, since social comparison serves the function of self-enhancement and self-improvement (Buunk & Gibbons, 2007). Compared to males, females tend to view themselves as below others on many dimensions (Goethals et al., 1991); thus, they might be more motivated to engage in higher levels of social comparison, seeking out negative information about others on Facebook. A previous study (Stefanone et al., 2011) has found that females are more susceptible to making online comparisons, especially on the dimension of physical attractiveness. The male tendency towards social comparison was found to only partially mediate the relation between self-esteem and time spent on Facebook. It is possible that males feel a less urgent need to compare themselves to others and that young adults with low self-esteem might spend more time on Facebook for other reasons too, e.g., social compensation (McKenna et al., 2002).

Concerning the different modalities of use, we obtained an interesting result related to the use of Facebook for social interaction. In line with previous research (Tartaglia, 2016), we found a positive relationship between self-esteem and using Facebook for social interaction, but only for females. Young women with a positive self-evaluation seem to use this social network for managing relations by chatting with friends and commenting on other people’s statuses, links or photos and for expressing themselves by publishing photos, videos and statuses. Some authors have already found that females are especially interested in relationship maintenance through social media (Rosen et al., 2010), and socially active females with positive self-esteem likely use Facebook as an additional means to maintain their social network. This result seems to be consistent with the rich-get-richer or social enhancement hypothesis (Kraut et al., 2002). Interestingly, when we introduced the dimension of social comparison, the situation completely changed. In fact, the use of Facebook by females to socially interact was also influenced by a high tendency towards social comparison, which in turn was determined by low self-esteem. The relevance of self-expression for females may explain this indirect relation. Women are more prone to prioritize and compare themselves on the dimension of physical attractiveness based on online photos (Nesi & Prinstein, 2015; Stefanone et al., 2011). Females with low self-esteem likely have a stronger need to compare themselves to other women in order to reduce uncertainty about the self and increase their self-esteem and positive affectivity (Buunk & Gibbons, 2007). For such girls, expressing themselves on Facebook might be a way to improve their self-evaluation. In sum, high and low self-esteem may increase women’s social interactions on Facebook for different reasons.

In line with previous research (Tartaglia, 2016), low self-esteem was related to the use of Facebook for simulation, but only for males. These data support the hypothesized compensatory use of Facebook for people with poor social skills and well-being (McKenna et al., 2002). People with a low self-evaluation are more likely to control the information about themselves by selecting photos to post and writing false self-descriptions that present socially desirable images of themselves (Tartaglia, 2016). These people construct their identities online, displaying their idealized selves on Facebook, and these self-presentations have the goal of increasing their subjective well-being (Kim & Lee, 2011). It is possible that people with low self-esteem try to improve their well-being by presenting themselves as different from how they are in real life. Introducing the dimension of social comparison orientation, we found that the use of Facebook for simulating a different self-image was also influenced by a high tendency towards social comparison, which in turn was determined by low self-esteem. This path was found for both males and females. As we expected, social comparison orientation was related to the use of Facebook for simulation: people high in social comparison had a strong interest in what others feel and think (Buunk & Gibbons, 2007), and this worry about image can lead them to simulate their online image. Our finding sheds new light on the relation between self-esteem and a potentially harmful modality of using Facebook. Self-esteem seems to have an indirect effect on the use of Facebook for simulating, and this effect is mediated by a tendency towards social comparison. This result highlights the importance of this personality characteristic in understanding why people behave in a determinate manner in social networks: people who are more prone to social comparison are at higher risk of developing maladaptive coping strategies, such as the construction of a false self to improve their well-being (Tartaglia, 2016). The results were slightly different between males and females. For females, social comparison orientation fully mediated the relation between self-esteem and the use of Facebook for simulating, because there was no direct path between the two variables. Females with low self-esteem and a high tendency towards social comparison seem to simulate more on Facebook, probably in order to gain benefits from the online comparisons, thus increasing their self-esteem. Concerning males, a tendency towards social comparison was found to only partially mediate the relation between self-esteem and the use of Facebook for simulation. Also in this case, we may interpret this result to mean that males probably feel a less urgent need to compare themselves to others, and young adults with low self-esteem might simulate more online for other reasons too, such as those predicted by the social compensation hypothesis (McKenna et al., 2002). Indeed, it is possible that males with low self-esteem present themselves differently from how they are in real life in order to facilitate the development of their social networks, thus meeting their social needs online. This result seems to provide further evidence of the particular importance of comparison for females, which can lead them to engage in self-presentation in a selective manner.

In line with previous research (Tartaglia, 2016), self-esteem and social comparison orientation were not related to the modality of using Facebook to search for relations. Individuals who use Facebook for meeting new people do not seem to utilize this social network to compensate for a deficiency in self-esteem.

The present study has some limitations. The use of cross-sectional data could limit the evidence in support of the direction of the relationships between self-esteem, social comparison, and use of Facebook. Future studies should use longitudinal data or an experimental design for a more rigorous examination of causal relationships. Moreover, the investigation should be extended to other age groups: the age of Facebook users has shifted over the course of the network’s growth, and the number of older users has increased dramatically (Wilson et al., 2012). Given this trend, it is important to investigate how young people’s Facebook use is similar to or different from that of older users. Finally, future studies should investigate the psychological and social consequences of spending time on this social network, assessing the impact of the different modalities of using Facebook on subjective well-being. For example, if individuals with low self-esteem and a high tendency towards social comparison are more apt to use these sites to simulate a different reality online, will their use cause positive or negative effects on self-esteem and well-being? These suggestions should be the starting points for further research studies.

In sum, the present study confirmed the importance of individual traits in determining different social behaviours enacted online. In particular, our findings shed new light on the well-known relation between self-esteem and the use of Facebook, introducing the social comparison orientation as a mediating variable. The results suggest that individuals with low self-esteem are especially interested in social comparison, and this personality characteristic is related to a greater amount of time spent on Facebook, motivated by the search for information about others. Especially for females, but also for males, the tendency to make social comparisons is associated with the use of Facebook for simulating a different self-image from reality: people high in social comparison have a strong interest in what others feel and think, and this concern may lead them to simulate their online image. For females, the use of Facebook for social interaction seems to be related to different needs. Young women with a positive self-evaluation use Facebook as an additional means to maintain their social network, whereas girls with a high tendency to social comparison, determined by low self-esteem, express themselves on Facebook, to improve their self-evaluation. In general, the tendency towards social comparison on Facebook seems to be especially important for females, mediating the effects of self-esteem. This finding suggests that gender differences should be taken into consideration when investigating these online behaviours. Since social media use can imply some risks, when it is carried out in a maladaptive way, it is important to prevent these risks by understanding the underlying processes. Particular attention should be given to people with uncertain identities: the use of Facebook for these problematic individuals might lead them to increased emotional difficulties because the social network can facilitate a dysfunctional modality of coping, such as the construction of a false self.
